# Illuminating growth: Celebrating the life and legacy of Dr. Joanne Chory

**DOI:** 10.1093/plphys/kiaf455

**Published:** 2025-09-26

**Authors:** Judy Brusslan, Jitesh Kumar, Neeta Lohani, Pei Qin (Sabrina) Ng, Meenu Singla-Rastogi, Andrew Willoughby, Avilash Singh Yadav

**Affiliations:** Department of Biological Sciences, California State University, Long Beach, Long Beach, CA 90840-9502, USA; Department of Plant and Microbial Biology, University of Minnesota, Saint Paul, MN 55108, USA; Center for Precision Plant Genomics, University of Minnesota, Saint Paul, MN 55108, USA; Department of Biotechnology, Thapar Institute for Engineering and Technology, Patiala, Punjab 147004, India; Department of Plant Sciences, University of Cambridge, Cambridge, Cambridgeshire CB2 3EA, UK; Department of Biology, Indiana University, Bloomington, IN 47405, USA; Department of Biology, Duke University, Durham, NC 27713, USA; Weill Institute for Cell and Molecular Biology and Section of Plant Biology, School of Integrative Plant Sciences, Cornell University, Ithaca, NY 14853, USA

**July 25–26, 2025, Milwaukee, WI USA**

**“Be Bold”**

Joanne Chory (Fig. 1) has shaped plant biology research for the last 30 years with her creative, insightful, and rigorous work on brassinosteroids and auxin, as well as light, temperature and retrograde signaling. Dr. Chory used a simple genetic system, *Arabidopsis* seedlings grown under different light wavelengths and intensities, for forward, reverse, and suppressor genetic screens. She was also a warm person who enjoyed her colleagues and family. Dr. Chory was highly driven, and she motivated those who worked with her. She famously noted, “I don’t ease into anything.”

**Figure 1.**
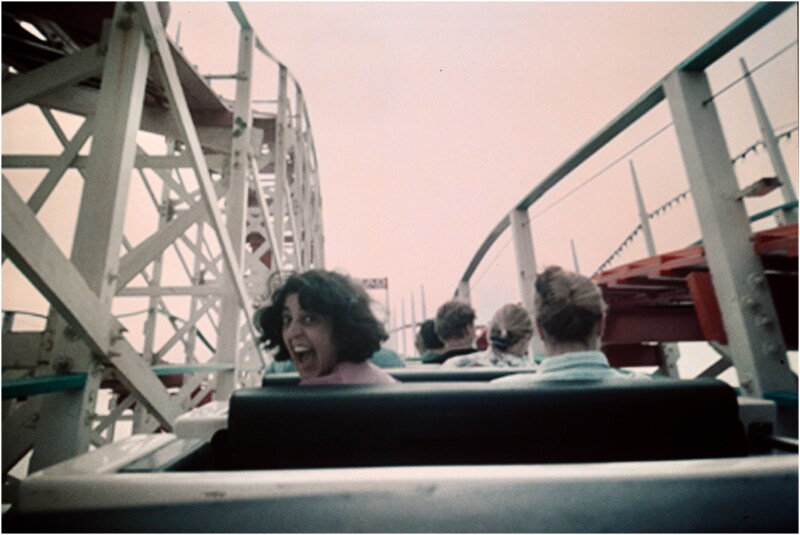
Joanne Chory on a roller coaster in 1995. Photo taken by Michael Neff, currently at Washington State University, when he was a postdoctoral fellow in the Chory lab.

The Joanne Chory Symposium featured talks by former (and current) members of Joanne Chory's lab, who are now leading plant biologists. Her guiding mantras, “Be Bold” and “Take risks, think big, and work on problems that matter,” were reflected in the exciting science and served to spark motivation for audience members who had not worked with her directly. The symposium was divided into 5 sessions, each reported by different assistant feature editors from the *Plant Cell* and *Plant Physiology*.

**“Take Risks and Think Big”**

**Light and Temperature Signaling**

Avilash Singh Yadav and Neeta Lohani

**Christian Fankhauser** (University of Lausanne, Switzerland) reflected on how his postdoctoral training with Chory in the late 1990s instilled the scientific values of fearlessness and collaboration. He presented insights on phototropins, blue light photoreceptors that mediate hypocotyl (embryonic stem) bending toward the direction of light. Fankhauser's group found that mutants of the ABC transporter *ABCG5* (*abcg5*) lack intercellular air spaces in the hypocotyl. He showed that intercellular air spaces facilitate light scattering within the tissue, thereby steepening the phototropin activation gradient that is essential for the directional light responses. **Toshinori Kinoshita**, now a professor at Nagoya University, Japan, had established that the phototropins in guard cells activate the plasma membrane H^+^-ATPases via phosphorylation to promote stomatal opening. His team subsequently identified conserved phosph-Thr881as key for activation. Kinoshita also showed that overexpression of H^+^-ATPases enhances stomatal conductance and nutrient uptake in rice roots, leading to higher shoot biomass in field conditions. **Adam Seluzicki**, a current Chory lab member from the Salk Institute, showed that phototropin2 works together with CAMTA2 to promote the expression of *ENHANCED HYPOCOTYL BENDING 1* (*EHB1*), which encodes a calcium-binding C2 domain protein that negatively regulates flowering in the dark by binding to NPH3 at cooler temperatures. Interestingly, blue light disrupts this interaction to promote flowering.

Professor **Lin Li** from Fudan University, China, described how she met Chory in 2006, who motivated her to become a principal investigator. Li shared her investigations on the shade avoidance syndrome (SAS), which is triggered when plants perceive a decrease in the red to far-red light ratio under the canopy of another plant. Li discussed how rhizosphere microbiomes modulate SAS in *Arabidopsis thaliana*, wherein specific root-associated microbial strains, such as *Pseudomonas fluorescens* and *Root918*, suppress shade-induced hypocotyl elongation.

Currently at Cold Spring Harbor Laboratory, **Ullas Pedmale** shared how Chory inspired him to work on photobiology. Pedmale shared recent scRNA-seq findings on shade regulation of root architecture. He showed that the receptor-like kinase FERONIA regulates shade avoidance responses with elevated levels of FERONIA in atrichoblasts in the root transition zones, leading to reduced root elongation in shade.

**Eirini Kaiserli** from the University of Glasgow, Scotland, did her postdoctoral training with Chory, an experience she described as being intellectually stimulating and collaborative. Kaiserli described her work on the conserved nuclear protein TANDEM ZINC-FINGER PLUS3 (TZP), which interacts with phytochrome B (phyB) and forms biomolecular condensates. Her group showed that TZP activates the expression of *CONSTANS* (*CO*) and *FLOWERING LOCUS T* (*FT*) while downregulating the floral repressor *FLOWERING LOCUS C* (*FLC*). She also showed that *tzp* mutants exhibit increased hypocotyl elongation and leaf area at elevated temperature, indicating TZP acts as a hub to integrate light, temperature, and flowering cues.

The final speaker of the session, Dr. **Joshua Gendron** from Yale University, reflected on how his interactions with Chory during his undergraduate years motivated him to pursue a PhD in plant biology. Gendron shared his recent findings on a long-day induced gene *MIPS1* (*MYO-INOSITOL PHOSPHATE SYNTHASE 1*), which encodes an enzyme that catalyzes the conversion of glucose to myo-inositol. Interestingly, *mips1* mutants exhibited severe vegetative growth defects in long- but not short-day photoperiods. His team showed that *MIPS1* function is governed primarily by the duration of photosynthesis ensuring sufficient carbon supply for growth.

**Organelle Signaling and Dynamics**

Meenu Singla-Rastogi

The session on *Organelle Signaling and Dynamics* brought together new insights into how plant organelles, particularly chloroplasts, signal, adapt, and coordinate with one another during development and stress. The presenters highlighted how chloroplasts operate as both metabolic engines and sophisticated signaling hubs.

The work presented by **Asa Strand** from Umea Plant Science Center reminds us that chloroplast biogenesis is not simply a matter of turning on photosynthetic genes. Instead, it requires a carefully timed sequence of molecular events. The plastid protein GUN1 functions as a safeguard, ensuring photosynthesis does not commence before the chloroplast is ready. This kind of checkpoint control is reminiscent of fail-safes in cell cycle regulation. The discovery that changes in histone modifications, transitioning chromatin from a closed to an open state, underlie the greening process further highlights that epigenetic control, related to GUN1 activity, is a critical layer of plastid–nuclear integration. Currently, at the Salk Institute, **Xuelin Wu's** findings illustrate how iron is directly involved in chloroplast development. The positive regulation of ferric-chelate reductase genes by STIP/WOX9 ensures iron reduction and delivery to the chloroplast and places iron metabolism squarely within the signaling framework of organelle development.

**Jesse Woodson** from the University of Arizona addressed how plants maintain chloroplast integrity under photooxidative stress. Chloroplasts can signal for their own degradation through a nonautophagic process for which the E3 ubiquitin ligase PUB4 acts as a central regulator. Transcriptome analyses showed that singlet oxygen increases jasmonic acid (JA) and salicylic acid (SA) stress hormones, but the increase in JA was reversed in *pub4* mutants. Distinctly, the semidominant *pub4* mutant showed constitutively high SA levels suggesting a critical role in keeping SA levels low. Finally, **Jianping Hu** from Michigan State University described a cytosolic shunt for photorespiration. Photorespiratory hydroxypyruvate reductase (*hpr1*) mutants are small and chlorotic. Hu's group employed a lovely example of Chory genetic strategy, and a suppressor line (*shpr7*) was identified that encoded a cytosolic glyoxylate reductase (GLYR1) that is part of a novel cytosolic photorespiration shunt that operates under higher light intensity.

**Auxin and Shade Avoidance**

Jitesh Kumar

**Jennifer Nemhauser**, currently a professor at the University of Washington, is addressing a longstanding question in plant biology: how the small molecule auxin can generate diverse yet highly specific responses. To dissect this complexity, her team recapitulated the auxin response pathway in *Saccharomyces cerevisiae* (yeast), enabling the study of auxin response circuits. This synthetic system has revealed critical insights into the degradation dynamics of Aux/IAA repressors in combination with specific Auxin Signaling F-Box (AFB) receptor family members. One key finding was the role of the transcriptional corepressor AtTOPLESS (TPL) in auxin signaling. Her work demonstrated that TPL not only interacts with the core mediator complex but also facilitates preassembly of the transcriptional machinery, enabling a rapid transcriptional switch from repression to activation. Dr. Nemhauser noted that auxin functions as a timer, primer, and coordinator, orchestrating precise developmental responses through both signaling dynamics and transcriptional control.

**Jason Reed** from the University of North Carolina Chapel Hill identified ARF6 and ARF8 as key mediators of auxin-induced flower and fruit maturation. His work revealed that auxin triggers gene activation postfertilization to promote fruit growth. *Arabidopsis arf6/8* double mutants had reduced fruit size even when fertilized with wild type (WT) pollen. Tomato plants with reduced *SlARF8A/B* expression were seedless and produced higher fruit yields in field trials.

**Carl Procko** from the Salk Institute presented on the role of auxin in stem elongation. Using the DR5::GUS auxin-responsive reporter, Dr. Procko demonstrated a redistribution of auxin signaling activity from the vasculature to the outer cell layers, specifically the apical tip of the hypocotyl following shade treatment. To dissect the tissue-specific requirements for auxin response, he expressed a stabilized mutant form of the Aux/IAA transcriptional repressor, axr3-1, in specific tissues. This approach allowed him to block auxin signaling locally. Notably, inhibiting auxin response in the epidermis significantly impaired hypocotyl elongation. Further, the findings suggest that the epidermis not only contributes to growth regulation through signaling but may also exert a mechanical constraint on inner tissue expansion. Supporting this idea, inner tissue layers exhibited increased growth when the epidermis was removed.

**Yogev Burko** from The Volcani Center in Israel has made higher order *pif* mutants in tomato and reported their hypocotyl growth responses to shade and warm temperature. Surprisingly, double mutants showed a greater shade avoidance response, while quadruple and septuple mutants had shortened hypocotyls. He also showed that growth in the hypocotyl is due to cell elongation, while growth in the epicotyl is due to cell division.

**Brassinosteroids**

Andrew Willoughy

**Jianming Li** from the University of Michigan started with the surprising figure that 30% of new proteins are misfolded and then proceeded to walk through his lab's work on using brassinosteroid receptor (BRI1)-based screens to identify new components of the ER-associated degradation (ERAD) protein control pathway, including unpublished work on how components required for ERAD are imported into the ER. **Zhiyong Wang** from the Carnegie Institution for Science presented work on crosstalk between BR and sugar signaling showing the BZR1 transcription factor is stabilized by sugar via Target of Rapomycin (TOR) signaling. He also showed that BSL1 phosphatase from *Chlamydomonas reinhardtii* (CrBSL1) is essential for mitosis, dephosphorylating CDLB1. **Grégory Vert** from the University of Toulouse showed his recent work on the post-translational modification and membrane targeting of BR1. BR perception is decreased by high temperatures because BRI1 is endocytosed and trafficked to the vacuole for degradation, and this is regulated by the action of the Desi3a SUMO protease that deSUMOylates BRI1.

**Sigal Savaldi-Goldstein** from the Technion in Israel focused on the functions of BR in shoots and roots and used grafting experiments to show that shoot BR is a limiting factor for root growth. Her RNA-seq data showed a starvation response in roots when BR production in the roots was lost. **Xuelu Wang** from Henan University in China discussed his current work on symbiotic nitrogen fixation in legumes. Utilizing large collections of soybean accessions, he used Genome-Wide Association Studies (GWAS) for nodule number to identify GmNNL1, an R gene. SINE insertion alleles in some accessions disrupt GmNNL1 allowing Bradyrhizobia to form symbiotic nodules. The session ended with **Takeshi Nakano** from Kyoto University in Japan presenting recent projects that focused on a gap in the canonical BR signaling pathway: how the transcription factor BRZ-INSENSITIVE LONG HYPOCTOYL1/BRASSINAZOLE-RESISTANT1 (BZR1/BIL1) and its homologs arrive to the nucleus to regulate transcription after BR perception. He showed that the newly identified BIL7 is a positive regulator of BZR1 nuclear translocation.

“**Work on Problems that Matter”**

**Harnessing Plants**

Pei Qin (Sabrina) Ng

The session “Harnessing Plants” beautifully encapsulates the late Dr. Joanne Chory's interests during the latter years of her career, which culminated in the establishment of the Harnessing Plants Initiative. At its core, this initiative is simple but ambitious: to leverage plants in addressing major global challenges. This session was led by **Yanhai Yin** (Iowa State University), **Elizabeth Fontes** (Federal University of Viçosa, Brazil), **Todd Michael** (The Salk Institute), **Björn Willige** (Colorado State University), and **Niko Geldner** (University of Lausanne, Switzerland).

The session began with an overview of scalable carbon capture using plants, 1 of 2 main goals of the Harnessing Plants Initiative. Michael's lab is now focused on screening plant varieties with ideal root architecture—deep and dense roots—to enhance carbon capture, known as the SALK ideal plant. Over the past 5 years, 22 SALK plants have been developed, 14 patents filed, and significant milestones reached toward modern agricultural carbon sequestration. Building on foundational research into BR, Yin's lab has expanded work in investigating the role of epigenetic signaling in BR biosynthesis. The understanding of epigenetic regulation of BR pathways will help improve crop yield and resilience, especially under drought stress, which is a major barrier to crop productivity.

The Fontes lab, in close collaboration with the Chory lab, has focused on unraveling the complex crosstalk between plant immunity and growth regulation via the nuclear shuttle protein-interacting kinase (NIK) signaling pathway. The latter part of the session shifted to plant–bacterium interactions, with the Willige lab characterizing an endophytic bacterium that promotes plant growth and the Geldner lab investigating mechanisms of bacterial root colonization at high spatial resolution.

Overall, the session's talks demonstrated an integration of Dr. Joanne Chory's research legacy and her profound influence on a new generation of scientists, as reflected in her words about “Harnessing Plants”:

*If we can optimize plants’ natural ability to capture and store carbon, we can develop plants that not only have the potential to reduce carbon dioxide in the atmosphere but that can also help enrich soils and increase crop yields.*

“**Genetics Is a Main Driving Force in Biology”**

**ASPB-Carnegie Winslow Briggs Mentorship Award**

Joanne Chory was also the corecipient of the inaugural **ASPB-Carnegie Winslow Briggs Mentorship Award**, and 3 Chory lab members spoke during the mentorship award session at the Plant Biology 2025 conference. **Meng Chen**, a professor at the University of California Riverside, reflected on his postdoctoral project to initiate the study of photobodies: phyB-containing membraneless organelles. Meng's lab recently elucidated the function of photobodies as sequestration sites for the PIF5 transcription factor, to titrate its transcriptional output in the nucleoplasm. His team also revealed the nonrandom, temperature-dependent photobody nucleation at distinct subnuclear locations. **Marco Bürger** from the Chory lab focuses on strigolactone receptors in root-parasitic parasitic plants, such as *Orobanche* and *Striga*. He investigates the structural basis of receptor binding to different classes of strigolactones. He developed high-throughput assays to identify agonists and antagonists for strigolactone receptors from different parasitic plant species, which have identified new inhibitors of parasitic seed germination. **Yunde Zhao** from the University of California San Diego shared Joanne Chory's advice on “Genetics is a main driving force in biology” and presented his discovery of a new type of genetic interaction called haplo-complementation. Disruption of *PIN1* or *PID* leads to the development of pin-like inflorescences and the failure of flower initiation. However, *pid* phenotypes are suppressed when *pin1* is heterozygous, whereas *pid* phenotypes are enhanced when *pin1* is homozygous. The discovery paved the way for studying the mechanisms by which auxin regulates flower development. Yunde knew that Joanne would be proud of the elegant genetic work he presented!

Although Joanne Chory was deeply missed at Plant Biology 2025, her insightful and creative ability to interpret genetic findings was well represented by those who worked with her and the cohort of plant biologists who read her papers. ASPB proudly supports the legacy of this singular plant biologist.

## Data Availability

The data underlying this article will be shared on reasonable request to the corresponding author.

